# Supplementary material for: Complex‐centric proteome profiling by SEC‐SWATH‐MS
Source: Mol Syst Biol. 2019 Jan 14;15(1):e8438. doi: 10.15252/msb.20188438 (PMC6346213; doi:10.15252/msb.20188438)
Supplement: Supplementary file 7 — Dataset EV6 [file MSB-15-e8438-s007.zip › feature_plots_bioplex/Q92665.pdf]

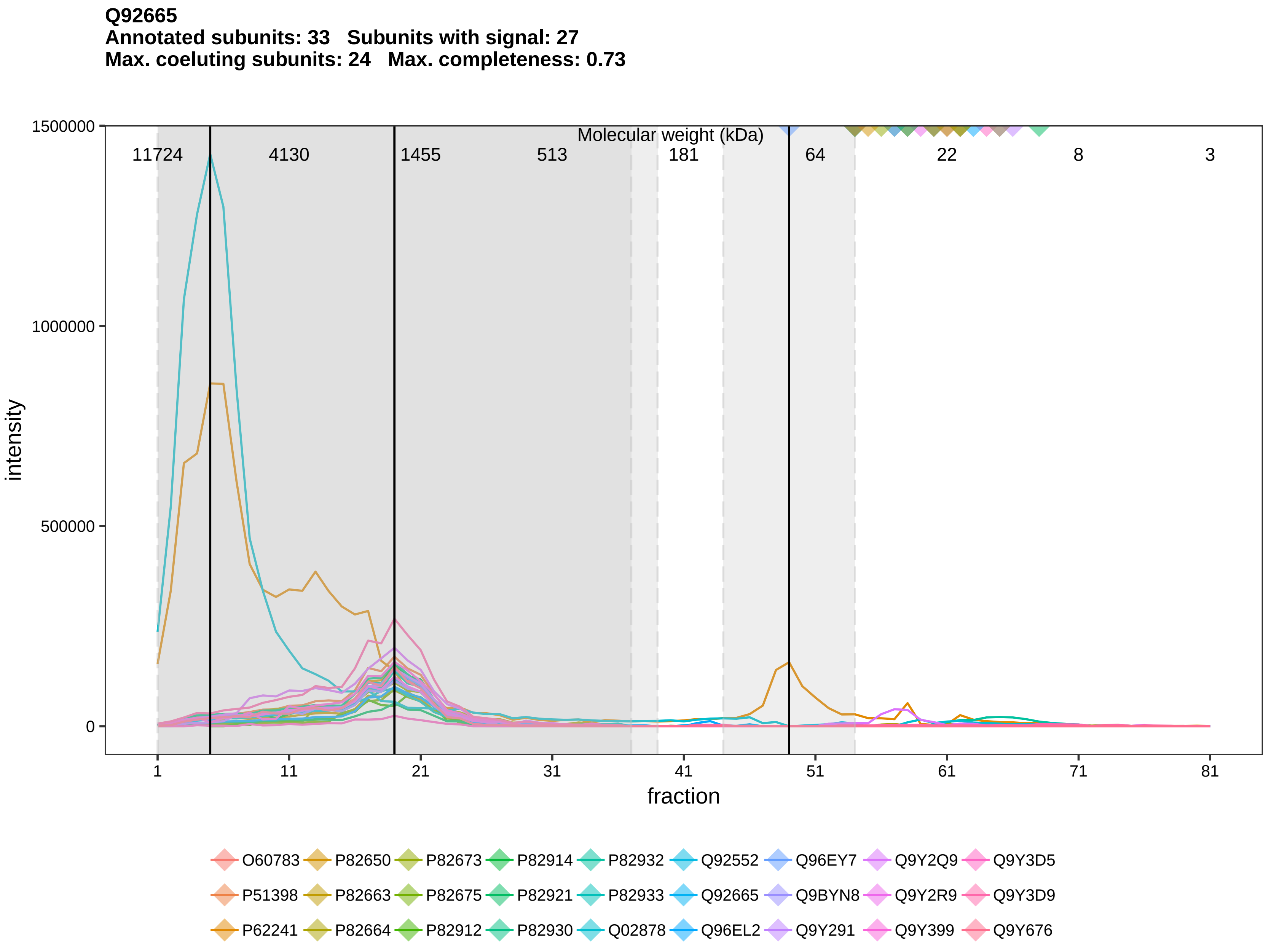

Q92665
Annotated subunits: 33   Subunits with signal: 27
Max. coeluting subunits: 24   Max. completeness: 0.73
Molecular weight (kDa)
11724
4130
1455
513
181
64
22
8
3
1500000
1000000
500000
0
intensity
1
11
21
31
41
51
61
71
81
fraction
O60783
P82650
P82673
P82914
P82932
Q92552
Q96EY7
Q9Y2Q9
Q9Y3D5
P51398
P82663
P82675
P82921
P82933
Q92665
Q9BYN8
Q9Y2R9
Q9Y3D9
P62241
P82664
P82912
P82930
Q02878
Q96EL2
Q9Y291
Q9Y399
Q9Y676
